# Editorial: Otitis media susceptibility due to genetic variants

**DOI:** 10.3389/fgene.2023.1341669

**Published:** 2023-12-12

**Authors:** Tal Marom, W. Edward Swords, Regie Lyn P. Santos-Cortez

**Affiliations:** ^1^ Department of Otolaryngology-Head and Neck Surgery, Assuta Ashdod University Hospital, Faculty of Health Sciences, Ben Gurion University of the Negev, Ashdod, Israel; ^2^ Department of Medicine, Division of Pulmonary, Allergy, and Critical Care Medicine, University of Alabama, Birmingham, AL, United States; ^3^ Department of Otolaryngology-Head and Neck Surgery, School of Medicine, University of Colorado Anschutz Medical Campus, CO, Aurora, United States

**Keywords:** Otitis media, cholesteatoma, gene, genetic susceptibility, middle ear, mouse model

## Introduction

Otitis media (OM) remains an important public health problem, particularly in children <5 years old who are most susceptible to OM-related hearing loss, which could affect their developmental milestones (GBD 2019 Hearing Loss Collaborators, 2021). While the incidence of acute OM markedly decreased during the COVID-19 pandemic (Marom et al., 2020), the increase of upper respiratory infections due to multiple otopathogens in the pediatric population after the relaxation of lockdown mandates suggests immune susceptibility (Kruizinga et al., 2023), part of which might be genetic in etiology (Bardou et al., 2020). Family history is a well-known risk factor for OM in its many forms (Huyett et al., 2018); however, compared to the epidemiology and microbiology of OM, genetic susceptibility to OM is less studied. Much of what we know about OM genetics relies on common variants from a few genome-wide association studies and several candidate gene associations of immune markers, rare variants in a few families, and gene-by-gene experiments in rodent models (Santos-Cortez et al., 2019; Giese et al., 2020).

## What this Research Topic contributes to scientific knowledge on Otitis media

For this Research Topic, we sought to pool research on various genetic models of OM. Five articles in this Research Topic describe the phenotypic changes and patterns of the course of OM in knockout mice or a novel humanized mouse model for OM (Kurabi et al.; Azar et al.; Kurabi et al.; Fons et al.; Son et al.). These mouse experiments present longitudinal data detailing the effects on the middle ear of the deletions of specific genes that play a role in middle ear structure or immune activation, which are difficult to document in humans. Taken together, these mouse models present snapshots of the complex host-bacterial interactions occurring in acute or chronic OM and in the future may be used for pre-clinical studies addressing specific components of the host response to OM.

In one human study by Lee et al., exome sequencing identified novel candidate genes—namely, *RTN4*, *RAB5A*, *CRYBG1*, *RGS22*, *APBB1IP*, *HEPHL1*, *BHLHE41*, *ARID3A*, *C5AR1*, *SPTLC3*, *CPT1B,* and *FAM227A*—for cholesteatoma in a single pediatric patient. Cholesteatoma is a cyst-like middle ear lesion that grows insidiously and erodes surrounding temporal bone structures, usually because of long-standing, inadequately treated chronic OM. Previous DNA exome and genome-wide association studies in humans have mostly focused on the common forms of OM, including acute OM, recurrent acute OM, chronic OM with effusion, and chronic suppurative OM (Santos-Cortez et al., 2019; Jamieson et al., 2021). Before this study, only two exome sequencing studies on families with cholesteatoma were published by the same group (Prinsley et al., 2019; Cardenas et al., 2023), with none of the candidate genes and pathways overlapping with those identified by Lee et al. These studies suggest that at least a subset of cholesteatoma patients has a potential genetic basis in their disease etiology. Of note, unlike acute OM, cholesteatoma is not as easily modeled in animals (Park and Lee, 2013), which makes the molecular characterization of human cholesteatoma an effective path to a better understanding of long-standing OM pathology.

## New mouse models for Otitis media

Two articles described the immune response in mouse models with known defects in their middle ear cavities. Azar et al. reported trans-cortical vessels in the tympanic temporal bone that connect the bone marrow to the middle ear mucosa lining the bulla cavity of *Mecom* ^*Jbo/+*^ and *Fbxo11* ^*Jf/+*^ mice, in which chronic suppurative or serous OM likely elicits a local neutrophilic rather than a systemic immunological response. In branchio-oto-renal syndrome 1, an autosomal dominant disease due to mutations in the transcription factor *EYA1* that mainly affects the ear as sensorineural hearing loss, it is also common for patients to have hypoplastic or malformed middle ears and OM (MIM 113650). Fons et al. reported in greater detail the middle ear defects in *Eya1* ^*+/−*^ mice, which have smaller otic bullae with remaining mesenchymal tissue within them, signaling a cavitation defect. Over time, the *Eya1-*mutant mice variably developed OM, as evidenced by inflammatory exudates, thickened mucosae, and disorganized ciliated and secretory cells. Interestingly, the cavitation delays in the *Eya1-*mutant mice were minimized in a germ-free environment.

Kurabi et al. presented the immune responses in *Ripk2* ^*−/−*^ and *Ercg4* ^*−/−*^ mice after bacterial inoculation with the common human otopathogen non-typeable *Haemophilus* influenzae (NTHi). The encoded Rip2 protein participates in the activation of the release of pro-inflammatory cytokines as part of the mucosal response to bacteria. In the single-cell RNA-sequence data from wild-type mouse middle ears, *Ripk2* was expressed in the epithelial, stromal, and endothelial cells at 6 h post-infection. In *Ripk2* ^*−/−*^ knockout mice, mucosal thickening and leukocytic infiltration were prolonged at 7–14 days compared to wild-type mice, with clearance of cells occurring late at 21 days in *Ripk2* ^*−/−*^ mice. On the other hand, *Ecrg4*, a regulator of tissue growth and macrophage activation that is expressed in middle ear stromal cells, was downregulated at 24 h post-inoculation. Additionally, *Ercg4* ^*−/−*^ mice had increased mucosal thickness, macrophage activity, and bacterial clearance at 3 days post-infection, indicating that anti-Ecrg4 might facilitate earlier resolution of OM through regulation of mucosal hyperplasia and leukocyte activity.

Son et al. developed a humanized mouse model that was engrafted with human stem cells and then infected with NTHi to induce acute OM. In contrast to mice that were not successfully engrafted with human stem cells, at 2 days after bacterial inoculation, the humanized mice developed mucosal hyperplasia, leukocytic infiltrates, and upregulation of inflammatory and immune response genes, while culture-verified recovery from OM occurred at 10 days post-inoculation, demonstrating immunocompetence. Immunohistochemistry confirmed the presence of human immune cells in the middle ear of the humanized mice. This new mouse model presents an exciting opportunity to model human OM, including cell-specific and time-dependent responses to different otopathogens and mimicking of the OM course in patients with various immune defects.

## Future perspectives

OM genetic studies in humans are useful in elucidating the mechanisms of OM susceptibility, particularly when genetic findings are supported by mechanistic findings in animal models with longitudinal and phenotypic data. Future studies may also include cell-based phenotypes due to infection and mutation as well as the change in virulence of otopathogens due to mutations.
